# Gut microbiota dysbiosis–induced chronic inflammation as a driver of atherosclerosis: cellular crosstalk and host–microbe interactions

**DOI:** 10.3389/fcimb.2026.1789194

**Published:** 2026-05-15

**Authors:** Da Song, Huize Gao, Tianqi Wang, Qian Wei, Aidong Liu, Jixiang Ren

**Affiliations:** 1 Changchun University of Chinese Medicine, Changchun, Jilin, China; 2 Department of Gastric and Colorectal Surgery, General Surgery Center, The First Hospital of Jilin University, Changchun, China; 3 The Third Affiliated Hospital of Changchun University of Chinese Medicine, Changchun, Jilin, China; 4 The Affiliated Hospital of Changchun University of Chinese Medicine, Changchun, Jilin, China

**Keywords:** atherosclerosis, chronic inflammation, fecal microbiota transplantation, gut microbiota dysbiosis, short-chain fatty acids, trimethylamine N-oxide

## Abstract

Gut microbiota dysbiosis is increasingly recognized as an upstream contributor to chronic low-grade inflammation and atherosclerosis (AS). Disruption of microbial homeostasis may impair intestinal barrier integrity, increase exposure to pro-inflammatory microbial products and metabolites, and reduce protective metabolites such as short-chain fatty acids (SCFAs), thereby activating innate immune signaling and sustaining vascular inflammation. Current evidence indicates that gut dysbiosis promotes atherosclerosis mainly through three interconnected processes: metabolite imbalance, barrier dysfunction with microbial translocation, and systemic immune reprogramming. Clinical studies have linked gut-derived biomarkers, particularly trimethylamine N-oxide (TMAO) and lipopolysaccharide (LPS)-related signals, to atherosclerotic burden and adverse cardiovascular outcomes, while experimental studies using fecal microbiota transplantation, probiotics, antibiotics, and gene-deficient models support a contributory role of the gut–immune–vascular axis. Emerging interventions, including dietary modulation, pharmacological repurposing, and microbiome-targeted therapies, may attenuate gut-derived chronic inflammation and offer new strategies for AS prevention and treatment. However, heterogeneity across studies and the limited causal evidence in humans warrant cautious interpretation. Overall, gut dysbiosis-driven chronic inflammation represents a biologically meaningful and potentially modifiable pathway in atherosclerosis.

## 1 Introduction

### 1.1 Disease burden of AS and unmet clinical needs

AS constitutes the pathological foundation of the vast majority of cardiovascular diseases (CVDs), which remain the leading cause of mortality worldwide. According to the Global Burden of Disease study, approximately 19 million deaths each year are attributable to CVD, nearly two-thirds of which are driven by AS (Roth et al., 2025). Although lipid-lowering therapies, including statins and PCSK9 inhibitors, together with antiplatelet strategies, have substantially improved cardiovascular outcomes (Jia et al., 2021; Wang et al., 2023a), the persistence of residual inflammatory risk underscores inflammation as a critical driver of AS progression that operates independently of lipid burden (Ridker, 2023). Landmark clinical trials such as CANTOS, COLCOT, and LoDoCo2 have provided compelling evidence that direct targeting of inflammatory pathways significantly reduces atherosclerotic events (Tardif et al., 2019). Nevertheless, concerns regarding the safety profile, long-term tolerability, and generalizability of current anti-inflammatory therapies have shifted research focus toward identifying upstream sources that sustain chronic inflammation.

### 1.2 Gut microbiota dysbiosis–driven chronic inflammation

Gut microbiota dysbiosis has emerged as a critical upstream driver of chronic low-grade inflammation (Fan and Pedersen, 2021). Rather than reflecting only a compositional shift in microbial taxa, dysbiosis also involves functional alterations in microbial metabolism, depletion of beneficial commensals, expansion of opportunistic pathobionts, and impaired production of anti-inflammatory metabolites (He et al., 2025). These changes weaken intestinal barrier integrity, facilitate the translocation of microbial-associated molecular patterns such as LPS, and promote persistent activation of innate immune pathways, thereby transforming local intestinal disturbance into systemic inflammatory stress (Violi et al., 2023). Recent studies further suggest that dysbiosis-associated chronic inflammation is sustained by a combination of barrier dysfunction, oxidative stress, altered host–microbe metabolite signaling, and maladaptive immune activation (Tonch-Cerbu et al., 2025).

**Figure 1:**
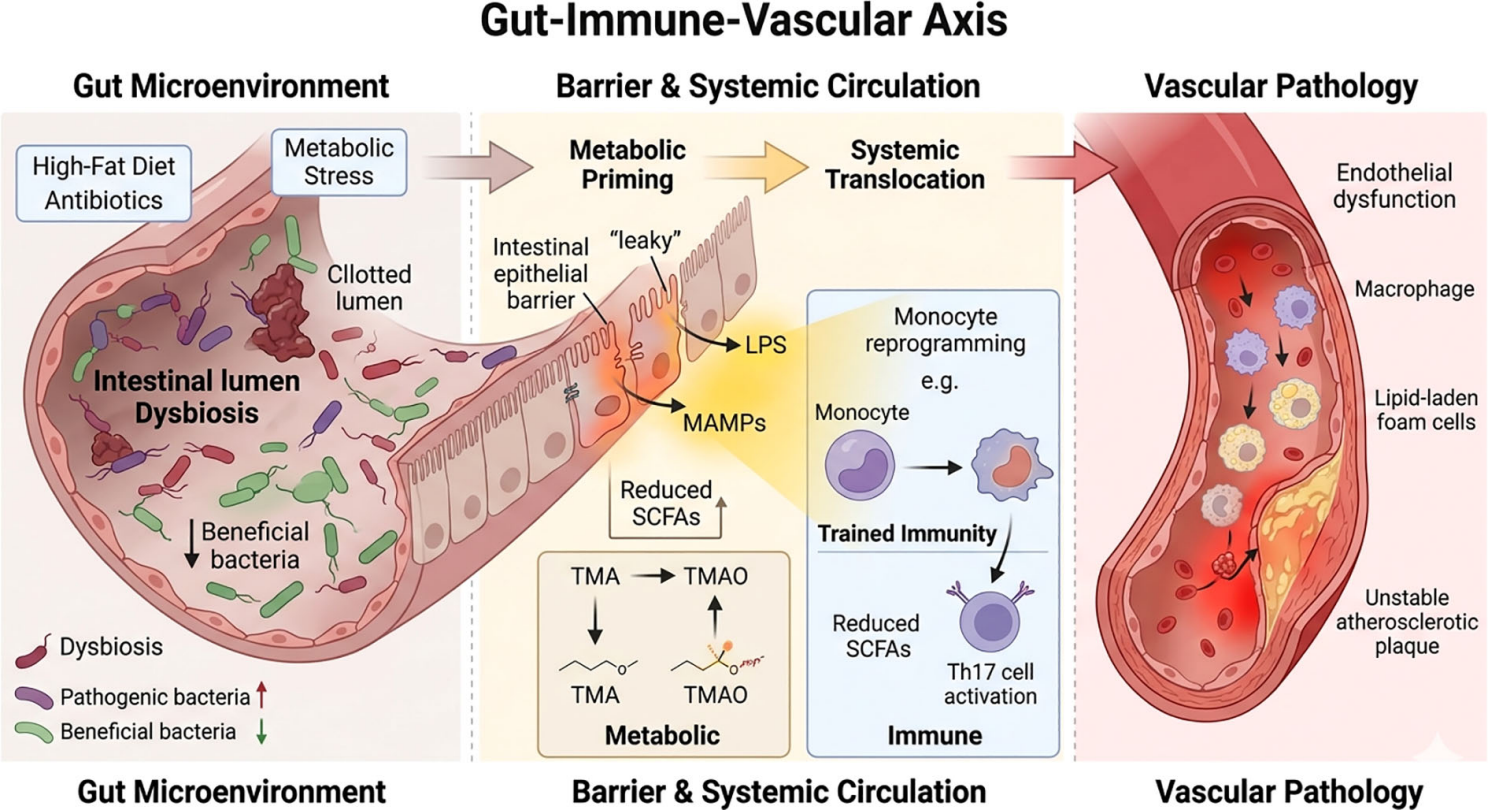
Conceptual framework of the gut dysbiosis–chronic inflammation–atherosclerosis axis. Environmental, metabolic, and disease-related stressors may disrupt gut microbial homeostasis, leading to loss of beneficial commensals, impaired barrier integrity, and altered metabolite production. These changes promote abnormal host exposure to pro-inflammatory microbial products and metabolites, including TMAO and lipopolysaccharide, and sustain immune reprogramming and chronic low-grade systemic inflammation. Through integrated effects on endothelial injury, foam cell formation, and plaque progression, this axis may contribute to the initiation and progression of atherosclerosis. TMAO, trimethylamine N-oxide; LPS, lipopolysaccharide.

In this context, the mechanistic framework can be conceptualized as four interconnected stages: microbial imbalance, barrier disruption, immune activation, and inflammation maintenance (Yeh et al., 2020). Through this process, persistent exposure to gut-derived inflammatory signals may generate an infection-like but non-classical inflammatory milieu, continuously activating innate and adaptive immune responses (Violi et al., 2023). This provides a biologically plausible basis for the proposed gut–immune–vascular axis linking intestinal dysbiosis to endothelial injury, vascular inflammation, and atherosclerotic progression (Witkowski et al., 2020). The specific molecular pathways and signaling networks involved are discussed in detail in Section 3.signalingsignaling. The overall conceptual framework illustrating the interactions between gut microbiota dysbiosis, chronic inflammation, and atherosclerosis is shown in **Figure 1**.

### 1.3 Scope and objectives of this review

Accumulating evidence supports a biologically plausible and increasingly well-substantiated association between gut microbiota dysbiosis–driven chronic inflammation and atherosclerosis, although the strength and causal interpretation of this relationship vary across biomarkers, study designs, and disease contexts. Circulating TMAO concentrations are closely correlated with AS prevalence, disease severity, and adverse prognosis (Zhao et al., 2023). Experimental studies further suggest that deliberate manipulation of the gut microbiota—through interventions such as fecal microbiota transplantation, probiotics, or antibiotics—can modify atherosclerosis-related inflammatory and vascular phenotypes in preclinical settings (Gregory et al., 2015). Collectively, these findings position the “gut-derived inflammatory axis” as a promising conceptual framework for understanding gut–inflammation–AS interactions and for identifying potential upstream therapeutic targets. The aim of this review is to systematically synthesize current evidence, delineate underlying mechanisms, and evaluate emerging therapeutic strategies within this rapidly evolving field.

## 2 Links between gut microbiota dysbiosis–driven chronic inflammation and coronary atherosclerosis

### 2.1 Clinical and epidemiological evidence

An expanding body of clinical evidence indicates that gut microbiota–derived metabolites and related circulating biomarkers—most notably TMAO and LPS or its binding protein (LBP)—are strongly associated with the incidence, severity, and adverse prognosis of atherosclerotic disease in humans. These associations suggest a potential role for such markers in cardiovascular risk prediction and stratification (Kanitsoraphan et al., 2018). Among microbial metabolites, TMAO is the most extensively studied and is supported by robust epidemiological data linking it to atherosclerosis. Tang and colleagues first demonstrated that elevated circulating TMAO levels were significantly associated with an increased risk of major adverse cardiovascular events (MACE) (Tang et al., 2013). Subsequent large-scale cohort studies and meta-analyses further consolidated these observations, showing that each 10 μmol/L increase in TMAO concentration is associated with an approximately 7.6% higher risk of all-cause mortality (Schiattarella et al., 2017). More recently, a prospective study by Budoff et al. reported that higher TMAO levels were linked to features of vulnerable coronary plaques, implicating TMAO in plaque instability and progression (Budoff et al., 2025). The prognostic relevance of TMAO appears even more pronounced in specific high-risk populations. In patients with peripheral vascular disease, Senthong et al. measured fasting plasma TMAO levels in 935 individuals with peripheral artery disease (PAD) and found that those in the highest TMAO quartile had a 2.86-fold higher risk of all-cause mortality compared with those in the lowest quartile (Senthong et al., 2016). Importantly, incorporation of TMAO into traditional risk models significantly improved the prediction of future cardiovascular events, underscoring its incremental prognostic value (Zheng et al., 2019). In parallel, markers of bacterial translocation, particularly LPS and its binding protein LBP, provide more direct evidence linking gut barrier dysfunction to atherosclerotic progression (Verhaar et al., 2020). Gorabi demonstrated that even subclinical elevations in circulating LPS constitute an independent risk factor for atherosclerosis development (Gorabi et al., 2022). In a prospective study involving 516 high-risk individuals, participants with baseline LPS levels exceeding 50 pg/mL exhibited a threefold increase in the risk of incident carotid atherosclerosis (Wiedermann et al., 1999). Consistently, in a larger cohort of 2,568 participants, baseline serum LBP levels were significantly associated with future cardiovascular events (Wang H. et al., 2022). Moreover, studies in patients with atrial fibrillation have, for the first time in large populations, demonstrated an independent association between circulating LPS levels and the risk of major adverse cardiovascular events, thereby extending the clinical applicability of this biomarker (D et al., 2017). Taken together, current evidence supports a meaningful association between gut microbiota–inflammation axis biomarkers, particularly TMAO and LPS/LBP, and atherosclerotic risk. These findings highlight the potential value of such markers in cardiovascular risk stratification and in framing mechanistic hypotheses for future intervention studies. However, these associations should be interpreted cautiously, as circulating TMAO and LPS/LBP levels are influenced by renal function, diet, comorbidities, and analytical variation, and most available human studies remain observational rather than interventional.

### 2.2 Associations in specific high-risk populations

Beyond the general population, certain groups of patients with chronic diseases represent clusters with an extraordinarily high incidence of atherosclerotic cardiovascular disease (ASCVD) (Rosenblit, 2019). In the following subsections, the emphasis is placed on host contexts that amplify the gut–immune–vascular axis rather than on re-describing its core molecular pathways.

#### 2.2.1 Chronic kidney disease: uraemic toxins, inflammatory burden, and accelerated vascular calcification

Patients with CKD are at extraordinarily high risk for atherosclerotic cardiovascular disease, with cardiovascular mortality far exceeding that of the general population (Jankowski et al., 2021). This excess risk cannot be fully accounted for by traditional cardiovascular risk factors alone (Naami et al., 2024). In this setting, reduced renal clearance and uraemic stress interact with gut microbiota dysbiosis to amplify systemic exposure to gut-derived metabolites, most notably TMAO (Zou et al., 2024). Rather than introducing a distinct mechanism, CKD should be viewed as a cardiorenal host context in which the kidney’s failure to excrete microbial byproducts magnifies their pro-atherogenic potential (Tomlinson and Wheeler, 2017; Mehta et al., 2023). This accumulation directly promotes endothelial dysfunction, vascular calcification, and accelerated atherosclerotic progression within the high-pressure pathophysiological environment of renal failure (Zhang et al., 2020; Chen et al., 2024). Detailed molecular pathways underlying these effects are discussed in Section 3.

#### 2.2.2 Heart failure: intestinal congestion, barrier disruption, and amplification of inflammation

Atherosclerosis is the most common underlying aetiology of HF, whereas established HF may in turn intensify gut dysbiosis-related inflammatory stress, creating a self-perpetuating vicious cycle (Florek et al., 2025). Owing to intestinal venous congestion, hypoperfusion, and the resulting “congestive enteropathy,” HF represents a hemodynamic host context that facilitates the extensive translocation of bacterial products (e.g., LPS) into systemic circulation (Trøseid et al., 2020; Fountoulakis et al., 2025; Snelson et al., 2025). In this sense, HF is best viewed not as a separate mechanistic pathway, but as a condition that facilitates “metabolic endotoxaemia” and accelerates the destabilization of pre-existing atherosclerotic plaques (Zhang et al., 2024; Epelde, 2025). The resulting persistent systemic inflammation further drives vascular injury as discussed in the context of the gut–immune–vascular axis (Witkowski et al., 2020).

#### 2.2.3 Type 2 diabetes mellitus: synergistic acceleration by metabolic dysregulation, dysbiosis, and inflammation

T2D is widely regarded as a “coronary heart disease equivalent” and is characterized by the early onset and diffuse distribution of AS (Leon, 2015). Beyond hyperglycaemia and insulin resistance, T2D is accompanied by profound gut microbial alterations and impaired barrier homeostasis (Yang et al., 2021; Baars et al., 2024). Hyperglycaemic conditions directly impair intestinal tight junctions (Rahman et al., 2022), creating a metabolic background that facilitates host exposure to gut-derived inflammatory signals (Cani et al., 2007). Accordingly, T2D should be regarded as a high-metabolic-stress context in which the shared processes of dysbiosis and “metabolic endotoxaemia” are magnified, reinforcing the low-grade inflammatory state that links metabolic dysfunction to vascular vulnerability (Galicia-Garcia et al., 2020). The specific molecular networks linking these processes to immune dysfunction are delineated in Section 3.

#### 2.2.4 Summary

In summary, populations with CKD, HF, and T2D serve as particularly informative models for examining the association between gut microbiota–driven chronic inflammation and atherosclerosis, not because these conditions represent isolated disease entities, but because they share—and markedly amplify—the common pathological endpoint of atherosclerosis. Within these models, disease-specific stressors, including uraemia, intestinal congestion, and metabolic dysregulation, converge on a unifying mechanism: the exacerbation of gut microbiota dysbiosis and systemic inflammation. This convergence consistently drives an accelerated atherosclerotic process. These conditions should therefore be viewed less as distinct mechanistic pathways and more as disease contexts that amplify shared gut-derived inflammatory processes.

Collectively, these observations support considering gut microbiota–driven chronic inflammation as a shared upstream contributor and a potential therapeutic target across high-risk disease contexts.

### 2.3 Experimental evidence

#### 2.3.1 Fecal microbiota transplantation: transmissibility of atherogenic microbial phenotypes

FMT, which involves transferring the entire intestinal microbial ecosystem from a donor to a recipient, is one of the most widely used experimental approaches for testing the transmissibility of microbiota-associated phenotypes in preclinical studies. In a seminal study, Gregory et al. transplanted gut microbiota from donors harboring a pro-atherogenic microbial profile into germ-free mice, resulting in a marked exacerbation of atherosclerotic lesions in the recipients. This work provided direct evidence for the transmissibility of atherosclerosis susceptibility via the gut microbiota (Gregory et al., 2015). Subsequent investigations, including studies published in JIE, further demonstrated that transplantation of fecal microbiota from patients with atherosclerosis into mice led to increased serum low-density lipoprotein cholesterol (LDL-C) levels and profound alterations in microbial metabolite profiles, supporting the notion that AS-associated microbial communities exert disease-promoting effects (Feng et al., 2025). Additional compelling evidence arises from models beyond classical cardiovascular disease. In experiments examining cardiovascular responses induced by chronic apical periodontitis, transplantation of gut microbiota from patients or diseased animal models into healthy mice resulted in more severe atherosclerotic lesions in the recipients, indicating that microbiota perturbations can transmit atherogenic susceptibility even across distinct disease contexts (Gan et al., 2024). Notably, even stronger causal inference is provided by studies showing that transplantation of microbiota from donors exposed to exercise or dietary interventions—both known to favorably modulate gut microbial composition—significantly attenuated atherosclerotic progression and ameliorated vascular inflammation in recipient mice (Men et al., 2025). Collectively, these findings support causal involvement of the gut microbiota in atherogenesis in experimental models and suggest that microbiota-associated phenotypes may be transmissible and, to some extent, reversible. However, whether these observations can be translated into causal inference or therapeutic efficacy in humans remains uncertain because of donor dependence, recipient heterogeneity, engraftment variability, and the lack of trials assessing hard atherosclerotic endpoints.

#### 2.3.2 Antibiotic and probiotic interventions: modifiability of gut microbiota–driven chronic inflammation

A substantial body of evidence demonstrates that partial depletion of the gut microbiota with antibiotics or supplementation with specific probiotic strains can markedly influence key pathological features of atherosclerosis, thereby establishing that microbiota-guided inflammation is amenable to targeted intervention. For example, Garshick et al. reported that antibiotic-induced dysbiosis using tylosin—characterized by an increased Firmicutes-to-Actinobacteria ratio and reduced α-diversity—attenuated the beneficial effects of lipid-lowering therapy on atherosclerotic plaque regression. This impairment was reflected by a failure to effectively reduce pro-inflammatory CD68-positive macrophage infiltration within plaques (Garshick et al., 2021). In contrast, probiotic interventions have shown potential benefits in preclinical models and small clinical studies, although the available human evidence remains heterogeneous and is mainly limited to surrogate endpoints. Chan showed that administration of the multispecies probiotic formulation VSL#3(a high-potency probiotic mixture containing multiple strains of Lactobacillus, Bifidobacterium, and Streptococcus), containing eight lactic acid bacterial strains, to apolipoprotein E–deficient (ApoE−/−) mice significantly mitigated high-fat diet–induced vascular inflammation and reduced aortic atherosclerotic lesion area (Chan et al., 2016). These experimental findings are corroborated by clinical data. In a randomized controlled trial conducted by Szulińska., 12 weeks of multispecies probiotic supplementation in obese postmenopausal women resulted in significant improvements in vascular function and reductions in arterial stiffness, accompanied by decreases in inflammatory biomarkers such as tumor necrosis factor-α (Szulińska et al., 2018). Together, these studies suggest that microbiota-directed interventions can modulate atherosclerosis-related inflammatory and vascular phenotypes; however, their reproducibility, optimal formulation, and clinical efficacy in humans remain to be established.

## 3 Potential mechanisms by which gut microbiota dysbiosis–driven chronic inflammation influences atherosclerosis: from intestinal signals to vascular inflammatory networks

**Figure 2:**
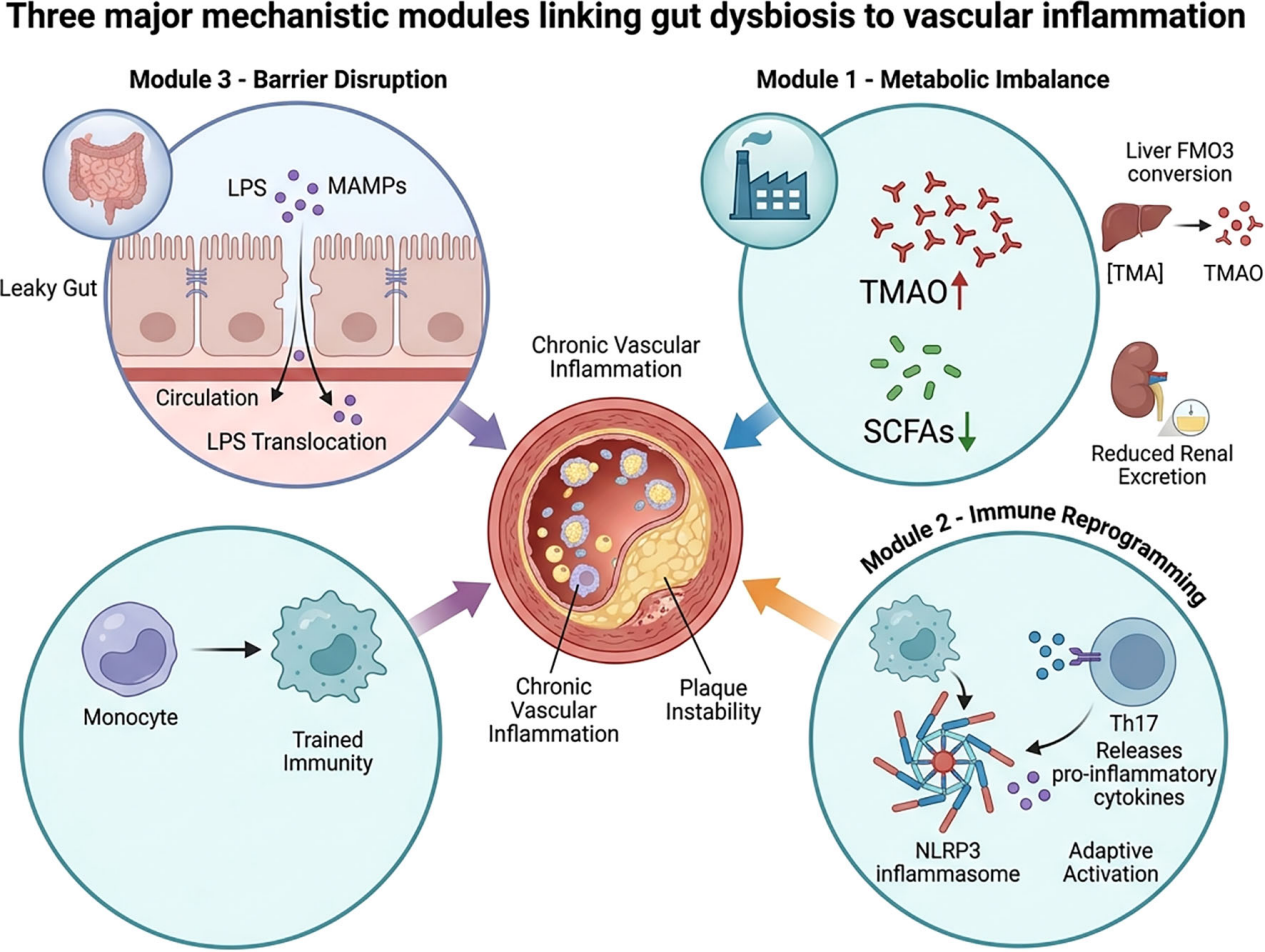
Three major mechanistic modules linking gut dysbiosis to vascular inflammation. Gut dysbiosis promotes atherosclerosis via three synergistic pathways: (1) Metabolic Imbalance, characterized by the accumulation of pro-atherogenic TMAO and loss of anti-inflammatory SCFAs; (2) Immune Reprogramming, involving the induction of innate “trained immunity” and sustained adaptive Th17 responses; and (3) Barrier Disruption, leading to the translocation of LPS and MAMPs into systemic circulation. These modules converge to amplify vascular inflammation, driving foam cell formation and plaque destabilization. TMAO, trimethylamine N-oxide; SCFAs, short-chain fatty acids; LPS, lipopolysaccharide; MAMPs, microbe-associated molecular patterns; Th17, T helper 17.

In the preceding sections, epidemiological studies and experimental interventions collectively support a biologically plausible relationship between gut microbiota dysbiosis–driven chronic inflammation and atherosclerosis. While several findings from animal and mechanistic studies suggest causal involvement of specific pathways, the strength and hierarchy of causality in humans remain context-dependent and incompletely resolved. Against this background, this section focuses on the molecular and cellular mechanisms through which gut-derived signals may contribute to vascular inflammation and atherogenesis. Gut dysbiosis influences vascular inflammation through three major mechanistic modules—metabolic imbalance, immune reprogramming, and barrier disruption—as illustrated in **Figure 2**.

### 3.1 Metabolic imbalance: direct “pro-inflammatory and pro-atherogenic” signals acting on the vasculature

While this subsection focuses on metabolite imbalance, barrier-mediated microbial translocation is addressed separately in Section 3.3. Gut microbiota–derived metabolites—most prominently TMAO—can directly activate pro-inflammatory pathways within vascular and immune cells, whereas SCFAs, particularly butyrate, exert protective effects via multi-target anti-inflammatory actions and barrier maintenance.

#### 3.1.1 TMAO (pro-inflammatory and pro-atherogenic actions)

Accumulating mechanistic and review evidence indicates that TMAO promotes atherosclerosis through multiple complementary pathways (Tang et al., 2013). On the one hand, TMAO upregulates macrophage scavenger receptors, such as CD36, thereby enhancing the uptake of oxidized LDL (Geng et al., 2018) and concomitantly suppresses reverse cholesterol transport, accelerating foam cell formation (Koeth et al., 2013; Warrier et al., 2015). On the other hand, in vascular endothelial cells, TMAO induces oxidative stress (Sun et al., 2016), activates NF-κB/MAPK signaling (Seldin et al., 2016; Boini et al., 2017), and triggers NLRP3 inflammasome activation (Chen et al., 2017; Zheng and He, 2022). These processes collectively promote inflammatory cell infiltration and vascular injury. Consistently, clinical studies demonstrate that elevated circulating TMAO levels are significantly associated with an increased risk of future MACE in patients with coronary artery disease (Wang et al., 2025). Taken together, TMAO may act as a mechanistic mediator in experimental models in addition to serving as a risk biomarker. However, its causal hierarchy and context-specific pathogenic contribution in humans remain incompletely resolved, particularly because circulating TMAO levels are strongly influenced by renal function, dietary exposure, and host metabolic status (Wang et al., 2011; Zhu et al., 2016).

In addition to metabolite imbalance, dysbiosis-associated microbial translocation represents another major route to vascular inflammation; this process is discussed separately in Section 3.3.signallingsignallingrecognise.

#### 3.1.2 SCFAs (failure of protective mechanisms):

In contrast to these pro-inflammatory signals, SCFAs are major metabolites produced by beneficial gut bacteria through fermentation of dietary fiber and exert pronounced anti-inflammatory, barrier preserving, and vasculoprotective effects (Du et al., 2024). SCFAs function as histone deacetylase (HDAC) inhibitors and activate G-protein–coupled receptors (GPR41/43/109A), thereby modulating metabolic and transcriptional programs in endothelial and immune cells (Guo et al., 2023). Through these mechanisms, SCFAs promote regulatory T-cell differentiation (Furusawa et al., 2013), suppress NF-κB activity (Chang et al., 2014), and enhance the expression of tight junction proteins such as ZO-1 and occludin, maintaining both intestinal and endothelial barrier integrity (Parada Venegas et al., 2019; Di Vincenzo et al., 2024). When butyrate-producing bacteria are depleted or dietary fiber intake is insufficient, SCFA levels decline (Liu et al., 2025), weakening host defensive mechanisms and rendering the vasculature more susceptible to injury by pro-inflammatory signals such as TMAO and LPS (El Hage et al., 2023). Emerging evidence further highlights the therapeutic potential of SCFAs in suppressing vascular inflammation and stabilizing the vessel wall (Zhao et al., 2025). Therefore, in the context of gut dysbiosis, vascular injury is accelerated not only by the amplification of pro-inflammatory signals but also by the concurrent failure of endogenous protective mechanisms.

### 3.2 Additional signaling pathways linking gut microbiota dysbiosis to chronic inflammation

In addition to the three core mechanistic modules outlined above, several signaling pathways function as important amplifiers or integrative nodes in gut dysbiosis–driven chronic inflammation. Rather than representing independent mechanisms parallel to metabolite imbalance, barrier dysfunction, and immune reprogramming, these pathways help explain how gut-derived perturbations are translated into persistent inflammatory activation and vascular injury. Among them, oxidative stress–inflammation coupling, renin–angiotensin system signaling, and aryl hydrocarbon receptor signaling have emerged as particularly relevant axes.

#### 3.2.1 Oxidative stress–inflammation coupling

Oxidative stress represents a major molecular amplifier of gut dysbiosis-mediated chronic inflammation (Sun et al., 2024). As discussed above, dysbiosis-associated metabolites such as TMAO can directly induce reactive oxygen species (ROS) generation in endothelial and immune cells, while depletion of protective metabolites such as SCFAs weakens antioxidant and anti-inflammatory defenses (Wang YN. et al., 2023). Beyond these metabolite-specific effects, oxidative stress may also be reinforced by persistent exposure to translocated microbial products and chronic immune activation. Excessive ROS not only impairs endothelial and epithelial barrier integrity but also activates redox-sensitive inflammatory pathways, including NF-κB and the TXNIP/NLRP3 inflammasome axis, thereby establishing a self-amplifying loop between oxidative stress and inflammation (Li et al., 2024; Wang et al., 2025). In this way, oxidative stress serves as a shared downstream effector linking gut dysbiosis to sustained vascular inflammatory injury.

#### 3.2.2 Renin–angiotensin system signaling

Another emerging mechanism is the interaction between gut dysbiosis-associated inflammation and the renin–angiotensin system (RAS). Although this pathway has not been as extensively characterized in atherosclerosis as TMAO or LPS related signaling, growing evidence suggests that gut microbial imbalance, barrier dysfunction, and endotoxemia may intersect with systemic and tissue-level RAS activation, particularly in cardiometabolic and cardiorenal disease settings (Jaworska et al., 2021). This interaction may be especially relevant in the CKD, HF, and T2D contexts discussed above, in which neurohumoral activation, oxidative stress, endothelial dysfunction, and chronic inflammation coexist (Schunk and Zimmermann, 2025). Increased angiotensin II signaling may further enhance NADPH oxidase activity (Kong et al., 2022; Zieleniewska et al., 2022), ROS generation, NF-κB activation, and vascular remodeling, thereby amplifying gut dysbiosis-driven inflammatory injury. Thus, the gut microbiota–RAS axis may function as a cross-organ inflammatory amplifier in susceptible host backgrounds.

#### 3.2.3 Aryl hydrocarbon receptor signaling

The aryl hydrocarbon receptor (AhR) is an important host sensor of microbiota-derived metabolites, particularly those generated from tryptophan metabolism, and serves as a key interface between microbial signals and host immune homeostasis (Dong and Perdew, 2020). Under physiological conditions, appropriate AhR activation supports epithelial integrity, mucosal immune balance, and IL-22-related barrier protection (Wang et al., 2023b; Wang K. et al., 2023). In the setting of gut dysbiosis, however, altered microbial metabolic output may reduce beneficial AhR ligands or disturb the overall ligand spectrum, thereby impairing barrier maintenance and favoring maladaptive inflammatory responses (Natividad et al., 2018). Through its crosstalk with oxidative stress and inflammatory signaling pathways, dysregulated AhR signaling may further contribute to persistent low-grade inflammation and downstream vascular injury. Therefore, AhR can be viewed as a mechanistic bridge linking altered microbial metabolism to barrier dysfunction, immune disequilibrium, and chronic inflammation within the gut–immune–vascular axis.

Collectively, these pathways do not replace the three core mechanistic modules described above, but rather provide additional molecular resolution for understanding how gut dysbiosis-derived signals are amplified, integrated, and sustained. By linking microbial metabolite imbalance, barrier dysfunction, and host inflammatory signaling, oxidative stress, RAS-related signaling, and AhR dysregulation help explain why chronic inflammation becomes persistent, systemic, and ultimately vascular-targeted in atherosclerosis. Against this background, long-term immune reprogramming represents a further step by which gut-derived inflammatory signals are maintained and directed toward the vessel wall.

### 3.3 Systemic immune reprogramming and cross-organ inflammatory crosstalk: persistence and vascular targeting of inflammation

Beyond direct effects of metabolites and microbial translocation, gut dysbiosis may also sustain atherosclerosis through long-term reprogramming of host immune responses. Gut-derived signals not only trigger acute inflammatory responses but also induce profound and sustained reprogramming of the host immune system (Tonch-Cerbu et al., 2025). This reprogramming converts transient intestinal perturbations into persistent, vascular-targeted chronic inflammatory attacks. Two key mechanisms underlie this process: trained immunity, which imparts inflammatory persistence, and lymphocyte homing, which confers inflammatory targeting. Trained immunity: Short-term or repeated exposure to gut-derived stimuli such as LPS or TMAO can induce long-lasting pro-inflammatory phenotypes in bone marrow progenitors and peripheral innate immune cells (Domínguez-Andrés et al., 2023). Mechanistically, this involves metabolic reprogramming, including upregulation of the mTOR-HIF- 1α axis (Zhong et al., 2020), and epigenetic remodeling, such as enrichment of H3K4me3 and H3K27ac at promoters of inflammatory genes (Fanucchi et al., 2021). Consequently, these immune cells respond more robustly and rapidly to subsequent atherogenic stimuli, such as oxidized lipids (Wang et al., 2023a). This mechanism explains the persistence and treatment resistance of vascular inflammation in AS, offering critical insight into the chronicity of the disease (Netea et al., 2020). Lymphocyte migration and local inflammation targeting: The gut is a central site for adaptive immune activation (Wang et al., 2024). Dysbiosis or altered metabolite profiles activate Th17 and Th1 cells, which may upregulate homing receptors such as CCR6 and CXCR3 and enter systemic circulation (Ma et al., 2022; Zheng et al., 2024). Studies suggest that these cells can respond to chemokines secreted by vascular cells in atherosclerotic lesions, including CCL20 and CXCL10, resulting in targeted migration to plaque regions (Aiello et al., 1999; Galkina et al., 2007). Once infiltrated, they secrete potent cytokines such as IFN-γ and IL- 17, directly amplifying local inflammation and destabilizing plaques (Kaden et al., 2003; Wolf and Ley, 2019). Collectively, systemic immune reprogramming and targeted lymphocyte migration driven by gut-derived metabolites establish a persistent, site-specific inflammatory loop, representing a central node in the gut–immune–vascular interaction (Fatkhullina et al., 2018; Mastrangelo et al., 2025).

### 3.4 Gut barrier disruption: the “source” for initiation and maintenance of inflammation

Loss of gut barrier integrity, manifested as increased intestinal permeability, serves as the key upstream source from which dysbiosis-associated inflammatory signals gain sustained access to the systemic circulation (Di Vincenzo et al., 2024). Barrier disruption permits translocation of gut contents—including LPS, microbial DNA, and metabolite precursors—into the circulation (Moludi et al., 2020; Violi and Nocella, 2023). These molecules function as persistent microbial-associated molecular patterns (MAMPs), activating pattern recognition receptors such as TLR4 and initiating downstream inflammatory pathways, thereby triggering systemic inflammatory responses (Stoll et al., 2004; Liu et al., 2017; Seethaler et al., 2021). Importantly, pathological states such as hyperglycemia or hypoperfusion can cause long-lasting downregulation of tight junction proteins (occludin, claudin-1, ZO- 1) and mucous layer damage (Sapone et al., 2006; Capaldo and Nusrat, 2009), facilitating chronic, low-dose translocation of MAMPs (Leech et al., 2019; Ghosh et al., 2021; Jaquez-Durán and Arellano-Ortiz, 2024). This chronic translocation sustains systemic inflammation (Violi and Nocella, 2023). Clinical evidence directly supports this mechanism: patients with coronary artery disease exhibit elevated circulating LPS and LBP levels, which are independently associated with more severe endothelial dysfunction and adverse prognosis (Lepper et al., 2011; Asada et al., 2019). Therefore, gut barrier disruption functions as both the initiator and maintainer of systemic chronic inflammation in atherosclerosis by enabling persistent translocation of pro-inflammatory microbial components (Lewis and Taylor, 2020).

### 3.5 Genetic evidence supporting innate immune mediation

Gene knockout models provide robust molecular-level evidence that gut microbiota dysbiosis promotes atherosclerosis primarily through innate immune pathways specialized in microbial sensing, rather than solely via metabolic or lipid-related mechanisms. Accumulating studies indicate that the pro-atherogenic effects of gut microbiota dysbiosis are not autonomous but critically depend on the activation of host innate immune pathways (Yeh et al., 2020).

#### 3.5.1 TLR4 signaling pathway

In atherosclerosis-prone mouse models, deletion of TLR4 reduces lesion burden, inflammatory signaling, and immune cell infiltration in the arterial wall, supporting the importance of microbial-sensing pathways in dysbiosis-associated vascular inflammation (Ding et al., 2012; Yu et al., 2025).

#### 3.5.2 NLRP3 inflammasome pathway

Likewise, genetic ablation of Nlrp3 attenuates lesion development and suppresses IL-1β- and IL-18-associated inflammatory activation, further supporting the requirement of innate immune mediation in microbiota-associated atherogenesis (Duewell et al., 2010; Mo et al., 2025).

Collectively, these knockout models highlight TLR4- and NLRP3-mediated innate immune sensing as central molecular bridges linking gut microbiota dysbiosis to chronic vascular inflammation. Experimental data from these models provide robust mechanistic support for the concept that chronic inflammation induced by microbial imbalance is a key driver of atherosclerosis, laying a solid theoretical foundation for future therapeutic strategies targeting these pathways to prevent or treat AS.

For clarity, the major mediators, mechanisms, evidence types, and principal limitations of gut microbiota dysbiosis-driven chronic inflammation in atherosclerosis are summarized in **Table 1**.

**Table 1:** Key mediators, mechanisms, evidence types, and major limitations of gut microbiota dysbiosis-driven chronic inflammation in atherosclerosis.

Key component	Major mechanisms	Clinical relevance to atherosclerosis	Evidence type	Limitations & controversies
TMAO elevation	Activates NF-κB- and NLRP3-related inflammatory pathways; promotes macrophage cholesterol accumulation, foam cell formation, endothelial dysfunction, and platelet hyperreactivity.	Positively associated with ASCVD risk, plaque burden, vulnerable plaque features, and major adverse cardiovascular events.	Cohort studies, meta-analyses, mechanistic studies, animal models.analyse	Causality remains debated: circulating levels are strongly influenced by dietary intake, renal clearance, and comorbidities; TMAO may act as both a pathway-associated effector and a disease-related biomarker.
Barrier disruption (LPS/MAMPs translocation)	Increased intestinal permeability permits translocation of LPS and other microbial-associated molecular patterns into the circulation, triggering TLR4-mediated innate immune signaling and systemic low-grade inflammation.signaling	Associated with endothelial activation, metabolic endotoxaemia, and increased cardiovascular risk in human biomarker studies.	Mechanistic studies, circulating biomarker studies, translational studies.	Measurement variability: lack of standardized assays for LPS/LBP in humans; substantial inter-individual variation and difficulty distinguishing source, timing, and biological activity.
SCFA depletion	Loss of GPR41/43 signaling and HDAC inhibition weakens epithelial integrity, anti-inflammatory signaling, and immune homeostasis.signaling	Associated with impaired barrier homeostasis, reduced anti-inflammatory buffering, and greater susceptibility to vascular inflammatory injury.	Dietary intervention studies, metabolomics, preclinical studies, mechanistic studies.	Confounding by diet: difficult to isolate the long-term effects of specific SCFAs from overall dietary fiber intake, host diet patterns, and broader metabolic context.fiber
Immune reprogrammingprogram	Induces trained immunity, metabolic/epigenetic rewiring of innate immune cells, and persistent adaptive immune activation, including Th17/Th1-skewed responses.	May contribute to persistent, treatment-resistant vascular inflammation, plaque progression, and inflammatory memory in atherosclerosis.	Genetic knockout models, translational immunology studies, mechanistic studies.	Translational gap: no standardized clinical metrics for measuring trained immunity or vascular immune memory in human atherosclerotic tissues.
Microbiome-targeted interventions (probiotics/FMT)	Reshape microbial composition and function, alter metabolite output, and potentially improve barrier integrity and inflammatory tone.	May improve surrogate inflammatory, metabolic, and vascular phenotypes and provide a potential microbiome-based adjunct strategy for atherosclerosis prevention or treatment.	Preclinical studies, small clinical trials, translational studies.	Inconsistent efficacy: intervention effects vary according to probiotic strain, donor effect, baseline microbiota composition, engraftment success, and study design; robust trials with hard cardiovascular endpoints are still lacking.

## 4 Targeting gut dysbiosis-driven chronic inflammation for the prevention and treatment of atherosclerosis: potential and strategies

### 4.1 Lifestyle and nutritional interventions

Lifestyle and nutritional interventions represent foundational and broad-spectrum strategies to modulate gut dysbiosis-driven chronic inflammation. Their benefits are realized through shaping dietary patterns, providing essential nutrients, improving metabolic status, and reducing systemic inflammation, all of which favor cardiovascular health. A healthy diet can robustly enhance beneficial microbial metabolites, such as SCFAs, strengthen gut barrier integrity, and diminish pro-inflammatory signals, thereby providing a crucial baseline for atherosclerosis prevention and management. The Mediterranean diet, rich in dietary fiber, polyphenols, and monounsaturated fatty acids, combines high plant-based food intake with abundant olive oil, nuts, and fish. This dietary pattern significantly increases gut microbial diversity and the abundance of SCFA-producing bacteria, such as Bifidobacterium and Roseburia (Cronin et al., 2021). These metabolites support gut barrier maintenance, reduce low-grade systemic inflammation, and improve metabolic profiles (García-Montero et al., 2021; Barber et al., 2023; Ratajczak et al., 2023). Studies demonstrate that adherence to a Mediterranean diet promotes SCFA production (Rodriguez et al., 2023), reduces pro-inflammatory bacterial populations, and improves gut function, ultimately lowering chronic inflammation and cardiovascular risk (Ghosh et al., 2020; Boughanem et al., 2023). SCFAs—including butyrate, propionate, and acetate—are the main fermentation products of dietary fiber by gut microbes (Fusco et al., 2023; Rasouli-Saravani et al., 2023). They serve not only as a primary energy source for intestinal epithelial cells but also modulate immune function and enhance the gut mucosal barrier through activation of G-protein-coupled receptors (GPR41/43) (Gasaly et al., 2021; Martin-Gallausiaux et al., 2021; Peng et al., 2022) and inhibition of histone deacetylases (HDACs). These actions suppress pro-inflammatory cytokines such as IL-6 and TNF-α (Randeni et al., 2024), contributing to reduced systemic inflammation (Liu et al., 2018; Kim, 2023). Multiple reviews and clinical studies support that high dietary fiber intake is associated with reduced circulating inflammation and improved cardiovascular risk factors (Primary prevention of cardiovascular disease with a mediterranean diet supplemented with extra-virgin olive oil or nuts, 2018; Papadaki et al., 2020; Kawaguchi et al., 2021), likely mediated by beneficial microbial metabolites and sustained metabolic improvement (Langfeld et al., 2021; Siddiqui and Cresci, 2021). Nutritional epidemiology also supports this link, highlighting fiber-mediated anti-inflammatory pathways as a key mechanism for reducing chronic inflammation (García-Montero et al., 2021). Dietary polyphenols, such as phenolic compounds in olive oil (hydroxytyrosol, catechins) and anthocyanins in berries, exhibit direct antioxidant properties and, importantly, are metabolized by gut microbes into more bioactive derivatives. These metabolites selectively promote beneficial bacteria, inhibit pro-inflammatory microbes, and reduce systemic inflammatory signals (Chacar et al., 2018; Marques et al., 2018; Mayta-Apaza et al., 2018; Wang et al., 2018b; The interactions between polyphenols and microorganisms, especially gut microbiota, 2026). Preliminary evidence links these polyphenol-derived metabolites with reductions in inflammatory markers and improvements in cardiovascular metabolism. Although numerous observational studies and short-term intervention trials support the beneficial effects of such dietary patterns on gut-driven chronic inflammation and cardiovascular risk markers, high-quality, long-term randomized controlled trials directly demonstrating causal effects on hard atherosclerotic endpoints (e.g., myocardial infarction, stroke) remain limited. Nevertheless, the current evidence indicates that optimizing dietary structure is a safe and broadly applicable foundational intervention, representing a key strategy for targeting gut-driven chronic inflammation to prevent and treat atherosclerosis (Randeni et al., 2024).

### 4.2 Drug repurposing

In recent years, several commonly used cardiovascular and metabolic drugs have demonstrated protective effects that extend beyond their classical targets, with emerging evidence linking these benefits to modulation of gut-derived chronic inflammation. These drugs may indirectly suppress systemic inflammation and atherosclerosis progression by improving gut ecology, reducing inflammatory signals, and regulating microbial metabolites, providing a novel mechanistic perspective for “drug repurposing” strategies.

#### 4.2.1 SGLT2 inhibitors

Sodium-glucose cotransporter 2 inhibitors (SGLT2i), such as empagliflozin and dapagliflozin, in addition to their canonical effects of reducing renal glucose reabsorption, improving glycemic control, and optimizing fluid balance, increasingly show potential in cardiovascular protection through modulation of the gut microbiome and inflammatory state (Kanbay et al., 2025). Preclinical studies report that SGLT2i promote the abundance of SCFA-producing bacteria, including Fecalibacterium prausnitzii and Bifidobacterium, concomitant with reductions in systemic inflammatory markers (Wang D. et al., 2022; Tariq et al., 2025). These effects are associated with improved gut barrier integrity and alleviation of metabolic endotoxemia (Liu et al., 2022; Afsar et al., 2024). Such microbiome-mediated improvements may represent a mechanistic basis for the extra-glycemic cardiovascular benefits of SGLT2i in diabetic and high-risk populations. Clinical studies further indicate that SGLT2i treatment reduces the relative abundance of pathogenic gut taxa, such as Fusobacterium, correlating with decreased inflammatory markers and improved endothelial function, suggesting that drug–microbiome interactions contribute to cardiovascular protection (Yang et al., 2025). Importantly, SGLT2i anti-inflammatory effects are not limited to shifts in microbiome composition; they also modulate immune cell programs, such as macrophage inflammatory responses (Pennig et al., 2019; Lee et al., 2020) and inflammatory signaling pathways including NLRP3 inflammasome and NF-κB (Sr et al., 2020), reinforcing their potential role as anti-inflammatory modulators in cardiovascular disease.

#### 4.2.2 Metformin

Metformin, a first-line therapy for type 2 diabetes, has long-established cardiovascular benefits in large clinical cohorts (Wimmer et al., 2024), yet its mechanisms extend far beyond improving insulin sensitivity. Substantial evidence shows that metformin significantly alters gut microbial composition, increasing the abundance of health-associated taxa such as Akkermansia muciniphila, and is associated with improved gut barrier integrity and reduced systemic inflammation (Petakh et al., 2023; Wimmer et al., 2024; Zhou et al., 2025). In prospective multi-omics analyses of coronary artery disease patients with diabetes, long-term metformin therapy was associated with increased abundance of beneficial microbiota, elevated anti-inflammatory metabolites, and reduced 5-year cardiovascular event risk, suggesting that modulation of gut metabolites and immune responses may improve atherosclerosis-related outcomes (Zhou et al., 2025). Experimental models demonstrate that metformin enhances SCFA production, decreases gut permeability, and reduces the translocation of exogenous inflammatory signals into circulation, thereby attenuating systemic chronic inflammation. These findings provide mechanistic support for its cardiovascular protective effects via the “gut–heart axis” (Wimmer et al., 2024). Taken together, metformin exerts cardiovascular protection through multiple pathways, including energy metabolism modulation and improvement of gut-derived inflammation. This multi-layered mechanism has attracted significant attention and represents a promising direction for future targeted research.

### 4.3 Emerging therapeutic approaches

**Figure 3:**
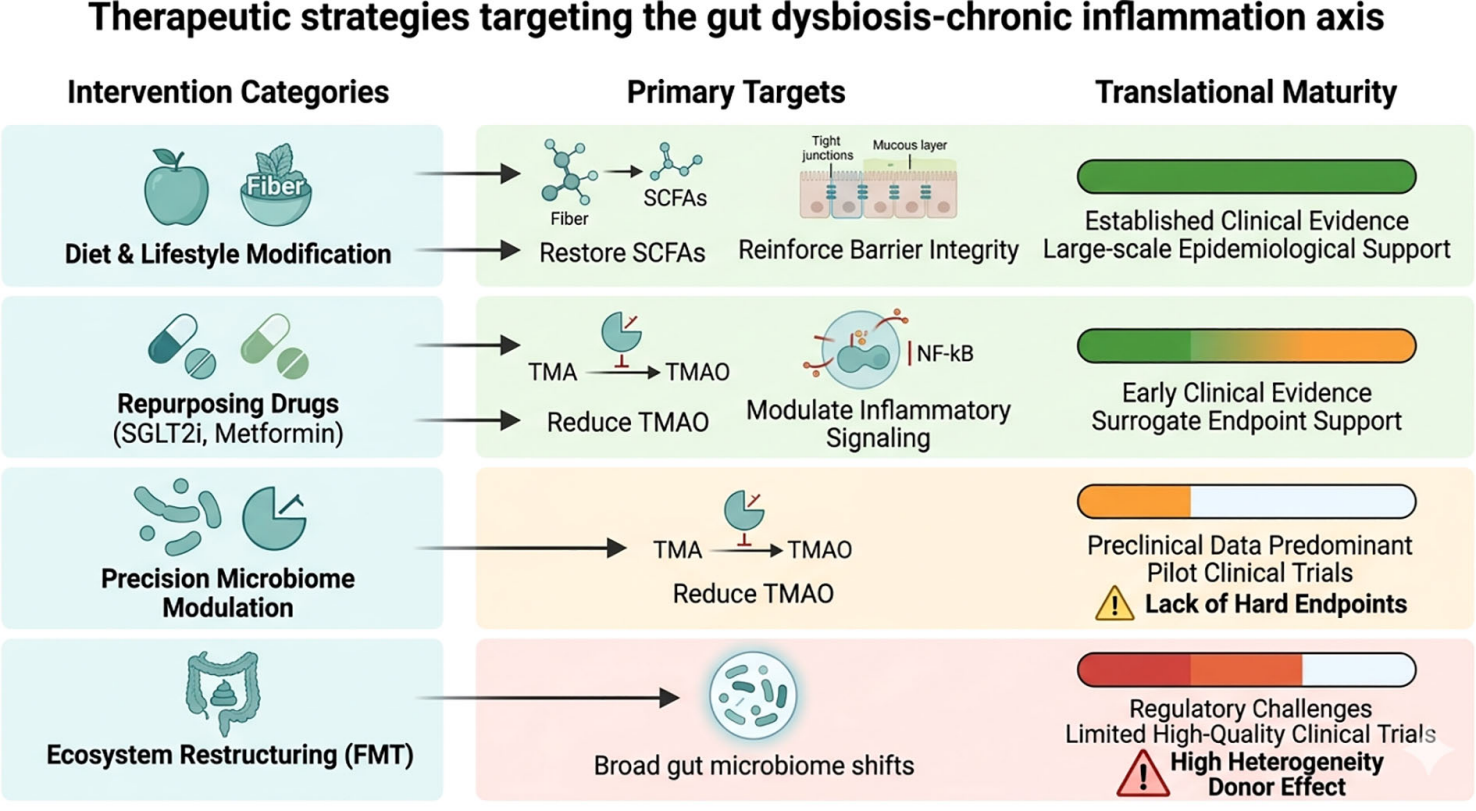
Therapeutic strategies targeting the gut dysbiosis–chronic inflammation axis: intervention level, primary target, and translational maturity. Current and emerging interventions target multiple levels of the gut–atherosclerosis axis, including lifestyle modification, pharmacological modulation, microbiome-targeted therapies, and ecosystem-level reconstruction. These strategies aim to reduce pro-inflammatory metabolites, restore barrier integrity, and modulate host–microbiota interactions. Their translational maturity varies substantially, with lifestyle interventions supported by robust clinical evidence, whereas precision microbiome therapies and fecal microbiota transplantation remain limited by heterogeneity, lack of standardization, and insufficient long-term clinical trials evaluating hard cardiovascular endpoints. FMT, fecal microbiota transplantation; TMAO, trimethylamine N-oxide.

A series of precise microbiome-targeted interventions are emerging at the forefront of cardiovascular disease prevention and treatment, aiming to mitigate chronic inflammation driven by gut dysbiosis. An overview of therapeutic strategies targeting the gut dysbiosis–chronic inflammation axis, including their intervention levels and translational maturity, is presented in **Figure 3**.

#### 4.3.1 Precision probiotics and prebiotics

Precision probiotic and prebiotic interventions are designed to supplement functional microbial strains or provide specific nutritional substrates to promote the growth of beneficial bacteria and enhance the production of anti-inflammatory metabolites, such as SCFAs, thereby improving intestinal inflammatory status (Zhou et al., 2025). Studies have demonstrated that various lifestyle, probiotic, and prebiotic interventions can partially ameliorate cardiovascular risk factors, including blood pressure (Yang et al., 2023), dyslipidemia (Wang et al., 2018a; O’Morain and Ramji, 2020), and inflammatory markers (Rajkumar et al., 2014), suggesting that targeted modulation of the gut microbiome may confer cardiovascular benefits.

#### 4.3.2 Microbial metabolic pathway inhibitors

Another emerging strategy is the direct inhibition of key microbial enzymes responsible for the production of pro-inflammatory metabolites, such as bacterial TMA lyase, thereby reducing harmful metabolites like TMA/TMAO that are closely linked to atherosclerosis (Roberts et al., 2018). Although these inhibitors remain at an early stage of human investigation, preclinical and translational studies have demonstrated their potential to markedly reduce pro-inflammatory mediators and mitigate the progression of atherosclerotic disease (Chen et al., 2025).

#### 4.3.3 FMT

FMT represents a holistic approach to reshaping the gut microbiome and has shown preliminary evidence of improving insulin sensitivity and metabolic status in studies on metabolic syndrome (Kootte et al., 2017; Sung et al., 2017). Systematic reviews have highlighted that the therapeutic potential of FMT in cardiometabolic interventions requires further evaluation through large-scale randomized controlled trials in humans (Leshem et al., 2019). Nevertheless, FMT provides a theoretical basis for targeting gut-origin chronic inflammation from an “ecosystem-level” perspective (Kang et al., 2017). These precision microbiome-targeted strategies intervene at multiple levels, including microbial composition, metabolite biosynthetic pathways, and overall ecosystem architecture, to modulate upstream inflammatory drivers of atherosclerosis. They represent a promising adjunct to conventional cardiovascular therapies. However, the definitive impact of these interventions on hard atherosclerotic endpoints remains to be established through larger, long-term randomized clinical trials.

## 5 Conflicting evidence, limitations, and possible explanations

Although a growing body of epidemiological, experimental, and translational research has supported the biological relevance of the “gut dysbiosis–chronic inflammation–atherosclerosis” axis in recent years, substantial uncertainty and controversy remain in this field. First, key metabolites such as TMAO have shown consistent associations with the risk of ASCVD in observational studies, yet their causal role in humans has not been fully established (Amaritei et al., 2025). Second, marked heterogeneity exists across human microbiome studies, and such variation arises not only from host background and environmental influences, but also from differences in study design, sequencing technologies, and endpoint selection (Motte et al., 2025). Third, although probiotics and FMT have shown potential benefits in animal models and some clinical studies, their efficacy in human populations remains clearly inconsistent (Battson et al., 2018). Taken together, these uncertainties suggest that current evidence more strongly supports this axis as a biologically plausible and clinically promising but complex regulatory network, rather than as a fully established linear causal pathway. In this context, cautious interpretation of the available evidence and systematic analysis of the sources of conflict are essential for moving the field from associative observations toward mechanistic understanding and effective intervention.

### 5.1 TMAO: association versus causality

TMAO is one of the most extensively studied gut-derived metabolites. Numerous cohort studies have shown that plasma TMAO levels are positively associated with the risk of ASCVD, plaque burden, and mortality. For example, in the Multi-Ethnic Study of Atherosclerosis (MESA), TMAO levels showed a dose-dependent association with cardiovascular events, with individuals in the highest quantile exhibiting a significantly increased risk (Budoff et al., 2025). At the same time, mechanistic studies indicate that TMAO may participate in atherosclerotic progression through multiple pathways, including promotion of foam cell formation, regulation of cholesterol metabolism, enhancement of platelet reactivity, and activation of NF-κB- and NLRP3-related inflammatory signaling, thereby aggravating vascular inflammation and plaque instability (Witkowski et al., 2020). However, despite the overall consistency of these findings at the experimental and epidemiological levels, important limitations remain in their causal interpretation in humans (Fan and Pedersen, 2021). First, TMAO levels are strongly influenced by multiple host factors, especially renal function (Obeid et al., 2024). Studies have shown that impaired renal function markedly reduces TMAO clearance, leading to substantially elevated circulating levels in populations such as patients with chronic kidney disease (Y et al., 2021). As a result, TMAO may function not only as a disease-related effector but also as a marker reflecting disease severity (Su et al., 2025). Accordingly, current evidence may support TMAO more strongly as a pathway-associated biomarker and candidate effector than as a universally established upstream causal driver in humans. After adjustment for renal function, dietary exposure, and comorbidity burden, the independent causal contribution of circulating TMAO in humans remains difficult to disentangle (Canyelles et al., 2023; Obeid et al., 2025).

### 5.2 Sources of heterogeneity in human microbiome studies

Although an increasing number of human studies support links between the gut microbiota and atherosclerosis-related metabolic abnormalities, inflammatory responses, and cardiovascular risk, findings across studies are often inconsistent. Such inconsistency does not necessarily indicate weak evidence in the field; rather, it may reflect systematic heterogeneity arising from differences in host metabolic status, concomitant medication use, model systems, and study design.

First, discrepant findings across studies of different drug classes suggest that results are highly dependent on clinical background and methodological conditions. For example, a recent review suggested that SGLT2 inhibitors may influence microbial composition, metabolite profiles, and inflammatory status (Kanbay et al., 2025), yet the currently available clinical and preclinical evidence remains heterogeneous, making it difficult to draw uniform conclusions. Such variation is likely related to key design variables. Across studies, participants differ in baseline hyperglycaemia, duration of diabetes, degree of obesity, renal function, and cardiovascular risk background, all of which can substantially influence the gut microbial ecosystem (Kanbay et al., 2025).

At the same time, concomitant medication use is another important but often underestimated source of confounding. Metformin itself can markedly alter the gut microbiome (Petakh et al., 2023). Therefore, when evaluating the microbiome-related effects of SGLT2 inhibitors, whether participants are also receiving metformin, antibiotics, or other metabolic drugs may substantially affect interpretation of the results. Previous studies have shown that metformin treatment can induce detectable microbial changes within a few months (P et al., 2023), and further work has emphasized that metformin should be regarded as an important confounder in studies examining the relationship between diabetes and the gut microbial ecosystem (Petakh et al., 2023).

In addition, differences in model systems may further amplify inconsistency across studies. Animal experiments can usually control diet, genetic background, and environmental exposure much more strictly, making microbiome changes easier to detect and interpret. In contrast, human studies are influenced by far more complex dietary patterns, lifestyles, medication histories, and comorbid conditions, so that the same intervention may lead to microbiome changes of different directions or magnitudes in different cohorts.

### 5.3 Why probiotic and FMT studies show inconsistent results

First, the interventions themselves are highly heterogeneous. Probiotics do not represent a single intervention, but rather a broad category composed of different strains, combinations, and dosages. Because different strains vary markedly in metabolic capacity, immunomodulatory effects, and colonization potential, direct comparison across studies is inherently difficult (Cruz Neto et al., 2024).

Second, the recipient’s baseline microbiota strongly influences treatment response. An increasing number of studies indicate that an individual’s initial microbial composition is a key determinant of whether probiotics or FMT can successfully engraft and exert their effects (Griffin et al., 2017; Kootte et al., 2017).

Third, FMT is characterized by a pronounced donor effect. Different donors possess substantially different microbial compositions, and the metabolic and immunological consequences after transplantation may therefore vary considerably. This donor dependency is one of the major reasons why FMT outcomes are often inconsistent (Kootte et al., 2017; Leshem et al., 2019).

Taken together, inconsistent efficacy of probiotics and FMT does not necessarily imply that these interventions are ineffective; rather, it more likely reflects their marked individual dependence and context specificity. Future studies should adopt more precise stratification strategies, such as patient grouping based on microbiome characteristics, standardize intervention protocols, and prioritize randomized controlled trials with hard clinical endpoints in order to better define the true clinical value of these approaches in atherosclerosis.

Overall, current evidence supports the biological and clinical relevance of the gut dysbiosis–chronic inflammation–atherosclerosis axis, but its causal hierarchy, dominant drivers, and clinically actionable subgroups remain to be clarified. The inconsistency across biomarkers, microbiome studies, and interventions suggests that this axis is highly host-dependent and context-specific. Future research should move beyond asking whether microbiome-targeted interventions work in general and instead identify which patient populations, microbial backgrounds, and mechanistic pathways are most likely to benefit.

## 6 Conclusion

Accumulating evidence from basic research, clinical association studies, and experimental interventions supports gut dysbiosis-driven chronic inflammation as an important link between host metabolic disturbances and the initiation and progression of atherosclerosis. Through integrated effects on barrier integrity, microbial metabolites, and immune reprogramming, this axis contributes to a sustained pro-atherogenic inflammatory milieu and may help explain the residual inflammatory risk that persists despite optimal control of traditional cardiovascular risk factors. Consequently, targeting gut-derived chronic inflammation represents a promising direction for future risk stratification and intervention development in atherosclerosis.

Several challenges remain before these insights can be translated into routine clinical application. The causal hierarchy of individual pathways remains incompletely resolved, and the strong influence of host genetics, long-term diet, drug exposure, and comorbidities contributes to marked inter-individual heterogeneity. In addition, findings from preclinical models require cautious validation in well-designed human cohort studies, interventional trials, and causal inference frameworks.

Future progress will depend on integrating mechanistic investigation with clinical validation. Multi-omics and systems-biology approaches should help move the field beyond correlative descriptions and clarify the dominant pathways linking dysbiosis, inflammation, and vascular injury across different stages of atherosclerosis. Clinically, priority should be given to identifying gut inflammation-related subgroups, refining biomarker-guided risk stratification, and testing microbiome-targeted interventions—such as dietary modulation, defined probiotic formulations, microbial enzyme inhibitors, and ecosystem-level approaches—in randomized trials with hard atherosclerotic endpoints.

In summary, gut dysbiosis-driven chronic inflammation should be regarded as a biologically meaningful and potentially modifiable contributor to atherosclerosis rather than as a fully resolved linear causal pathway. With continued integration of mechanistic insight, biomarker refinement, and precision intervention design, this axis may evolve into a clinically actionable framework for upstream cardiovascular.
